# Quality of life in patients with thalassemia major

**Published:** 2014-04-20

**Authors:** Sh Ansari, A Baghersalimi, A Azarkeivan, M Nojomi, A Hassanzadeh Rad

**Affiliations:** 1 Pediatric Hematologist- Oncologist, Department of the Pediatric Hematology and Oncology, St Ali- Asqar Hospital,Tehran University of Medical Sciences, Tehran, Iran.; 2 Pediatric Hematologist- Oncologist, Pediatrics growth disorders research center , 17 shahrivar hospital, school of medicine, Guilan University of medical sciences, Rasht, Iran.; 3 Pediatric Hematologist- Oncologist, Iranian BloodTransfusion Organization Research Center, Tehran, Iran.; 4 Professor of Community Medicine. Department of Community Medicine, School of Medicine, Tehran University of Medical Sciences. Tehran, Iran; 5 Pediatrics growth disorders research center, 17 shahrivar hospital, school of medicine, Guilan University of medical sciences , Rasht, Iran

**Keywords:** Quality of life, Thalassemia Major, WHOQOL- BREF

## Abstract

**Background:**

With modern medical management, thalassemia major is now extending into adulthood and it is expected to have a negative impact on the quality of life (QOL) of the patients. The aim of this study was to evaluate quality of life in patients with thalassemia major.

**Materials and Methods:**

This is an analytic case control study. Two hundred and fifty patients and 51 participants as controls were assessed using WHOQOL- BREF (Farsi version) questionnaire. All questions were answered based on the self-evaluated status in the past 2 weeks before enrollment and were rated on a five-point Likert scale. Therefore, the raw item score ranged from 1 to 5 and scaled in a positive direction and 6 dimensions including overall QOL, overall health, physical, psychological, social, and environmental relationship were assessed.

**Results:**

Results showed that the QOL in all 6 dimensions was lower in patients compared to the controls (P< 0.05).Also age, higher education level, lower ferritin level and using oral iron chelator were associated with better QOL scores. On the other hand, cardiac disease, hepatitis C and history of psychiatric disorders were associated with impaired QOL scores.

**Conclusion:**

These findings were important for future refinement of national thalassemia program. So, we recommended regular screening for psychiatric disorders and facilitated access to oral iron chelators. Regular monitoring and treatment of complications especially cardiac disease and hepatitis along with strict quality control of blood products were also mandatory. Also, higher education of the patients may improve quality of life.

## Introduction

Thalassemia as the most common genetic disorder worldwide is regarded as a serious problem in public health issues in the Mediterranean region (1). Iran is located in the geographical belt of thalassemia and it has been estimated that thalassemia carriers vary from one to ten percent (with a mean of 4.5%) in different parts of Iran (2).

Although, morbidity and mortality of the thalassemia major has been reduced significantly in the light of modern medical treatment, however, it could influence diverse aspects of patients’ lives. Some aspects of thalassemia major and its associated complications are expected to impact on the QOL. the diagnosis and treatment of the thalassemia major could have an impact on family stability and family dynamics and bone deformities and short stature could induce poor self-image. Also, frequent hospital visits for transfusion, nightly subcutaneous infusions, delayed or absent sexual development and impaired fertility and complications such as heart disease, bone disease, diabetes, infections and Uncertainties about the future and difficulties in long-term planning could be mentioned as a result of thallassemia major (3). The World Health Organization (WHO) defined the quality of life as : “An individual perception of their position in life in the context of the culture and value systems in which they live and in relation to their goals, expectations, standards and concerns. It is a broad ranging concept affected in a complex way by the person's physical health, psychological state, personal beliefs, social relationships and their relationship to salient features of their environment(4).

There are few inconsistent studies about the QOL in patients with thalassemia major and most of these investigations obtained the results based on interviews with patients, careers, doctors and nurses with the focus on coping strategies and they did not mention control group. Previous investigations showed that treatment and cultural differences did not have a major effect on the QOL (5). Also, results indicated that patients had moderately impaired overall health and overall QOL (6) and other serious hemoglobinopathies such as sickle cell disease (SCD) might induced poor QOL (7-10) However, there might be differences in the domains affected as well as the extent of variation across specific chronic disorders (11).

In the present study, we aimed to assess the Quality of life in patients with thalassemia major

## Materials and Methods

Patients and setting

This is an analytic case control study which was conducted on 301 participants (250 patients and 51 healthy people) referred to Clinic of st Ali-Asqar Hospital and Zafar Clinic, Tehran , Iran between December 2009 to September 2010. The inclusion criteria for patients were indicated as transfusion dependent (major) thalassemia and ≥18 years of age. Also, Exclusion criteria were any debilitating disorders unrelated to thalassemia major and its associated complications such as malignancy, seizure disease and mental disorders. Control group consisted of ≥18 year’s old healthy people with no mentioned medical history. Groups were matched based on age and sex.

Instrument

The WHOQOL- BREF (Farsi version)is an easy, self administer questionnaire which was used in this study (12).Based on administration guide manual of WHOQOL- BREF, It consisted of 26 questions which evaluates the QOL in 6dimensions (5) included: Overall QOL with one question, Overall quality of health with one question, Physical domain of the QOL with 7 questions, Psychological domain of the QOL with 6 questions, Social domain of the QOL with 3 questions, Environmental domain of the QOL with 8 questions. (12)

All questions were responded based on the self-evaluated status in the past 2 weeks before enrollment and were rated on a five-point Likert scale. Therefore, the raw item score ranged from 1 to 5 and scaled in a positive direction (i.e. higher scores denoted higher quality of life). Based on manual administration guide, after collecting the raw data, they were modified and the analysis was performed.

**Statistical analysis**

Demographic characteristics, disease features and disease associated complications were analyzed. T-test was used to compare mean of ages and quality of life domains in two groups and Chi-square test compared nominal explanatory variables. Spearman correlation coefficient was used to assess correlation between numeric demographic and clinical variables with domains of quality of life. Also, multiple linear regression analysis was used to assess independent association of each explanatory variable with domains of quality of life and variables with significant association in domains of quality of life were evaluated by bivariate analyses. Each domains of quality of life were considered as dependent variable, and demographic and clinical variables with significant associations were put as independent variables in separate six models. Statistical significance was considered as P- value < 0.05. This study was approved by the ethics committees of both clinics and consent letter was obtained from participants.

## Results

Results indicated that 121 female (48.4%) and 129 male (51.6%) participated in this study, 93 patients were unemployed (62.4%) and 48 patients were employee (32.2%). Table I showed demographic characteristics of participants.

The thalassemia major was diagnosed in 39.2% of patients under one year old (39.2%), and most of them (62.8%) used overnight subcutaneous infusion of Deferoxamine by a pump. About 56% of patients had been splenectomized. Although, the most common complication was hypogonadism (44.8%) but hepatitis B with frequency of 2.8%was less common. Table II, illustrated the disease-related features and prevalence of the thalassemia-associated complications in patients. 

Results demonstrated significant difference in all domains of QOL (P < 0.05) between groups.(Table III)

Overall quality of life. 

According to results, higher education level (rs= 0.23, P= 0.001) and lower ferritin level (rs= -0.10, p= 0.05) were associated with better overall quality of life during a week. Female patients had a better quality of life than males significantly (3.56, versus 3.36; p= 0.05). 

The presence of hepatitis C (3.22 versus 3.54; p=0.02), cardiac disease (2.91 versus 3.54; p= 0.001) and history of psychiatric disease (2.56 versus 3.49; p= 0.002) were negatively associated with overall QOL. Although unsplenectomized patients had better overall QOL but the difference was not significant. After adjusting all variables, educational level, hepatitis C infection, cardiac and psychiatric disorders had a significant association with overall quality of life (P< 0.01). 

Overall health

Lower ferritin level (rs= -0.16, p = 0.008), and female gender (3.48 versus 3.19; p = 0.02), were associated with better overall quality of health. Suffering cardiac disease (2.64 versus 3.44; p = 0.001) and hepatitis C (3.03 versus 3.44; p = 0.005) were associated with lower overall quality of health. By using multivariate analysis, the association of ferritin level, having hepatitis C, and cardiac disorders was significant. (P< 0.01).

Physical health

Lower age (rs = -0.15, p = 0.01) and higher educational level (rs = 0.19, p = 0.02) were associated with better physical health and having cardiac disease (55.18 versus 66.42; p = 0.001) was associated with lower physical health related QOL.

Educational level and cardiac disorders had a significant association with physical health domain after adjustment (P< 0.01).

Psychological health

Higher education level was associated with better psychologic health related QOL (rs = -0.18, p = 0.003) but hepatitis C (49.60 versus 57.08; p = 0.005) and cardiac disease (45.73 versus 56.57; p = 0.002) were associated with lower psychological health. Also, these variables were associated with psychological domain after adjustment (P< 0.01).

Social relationship

Lower age (rs = -.17, p = 0.008), higher education level (rs = 0.16, p= 0,01) and giving oral iron chelation compared to deferroxamine (61.71 versus 46.50, p = 0.009) were associated with better social relationship domain of the QOL. After adjustment, these associations were remained significantly different (P<0.05, P<0.01).furthermore, history of psychiatric disorder (46.44 versus 60.10; p = 0.05) was associated with lower social relationship. The association of psychiatric disorders with social relationship was not significant in multivariate analysis.

Environmental health

Higher educational level (rs = 0.23, p = 0.001) was associated with better environmental health related QOL. History of psychiatric disorder was associated with lower environmental health (36.78 versus 56.55; p = 0.002). These variables had an adjusted significant association with environmental health. (P< 0.01).

The compared domains scores of quality of life by demographic and clinical characteristics of patients had been shown in Table IV.

**Table I:** Demographic features of patients and controls

	**250 Patient** **No(%)**	**51Control** **No(%)**
**Sex** **Female** **male**	121(48.4) 129(51.6)	28(54.9) 23(45.1)
**Age(mean±SD) **	25.86 ±4.94yrs	25.00±5.50yrs
**Educational level** **-illiterate** **-secondary school and lower** **-diploma or bachelor of science** **- master of science and higher**	2(0.8) 48(19.2) 193(77.2) 7(2.8)	0 10(19.6) 39(76.5) 2(3.9)
**Marital status ** **-single** **- married** **-divorced**	200(80) 46(18.4) 4(1.6)	36(70.6) 15(29.4) 0

**Table II:** Serum levels of ferritin and cardiac T2*MRI

**Disease feature**	**No. (%)**
**Age of diagnosis** **<6 month** **<1year** **<2year** **2year and more**	48(19.2) 98(39.2) 49(19.6) 55(22)
**Interval of transfusion:** **2 weeks or less** **3weeks** **4weeks** **5weeks or more**	63(25.2) 118(47.2) 50(20) 19(7.6)
**Ferritin (ng/ml):** **<500** **500-999** **1000-1999** **2000-2999** **>3000**	35(14) 48(19.2) 83(33.2) 36(14.4) 48(19.2)
**Chelator :** **Deferroxamine** **Defrasirox** **Combined ** **Unknown**	157(62.8) 21(8.4) 42(16.8) 30(12)
**Splenectomized**	139(55.6)
**Complication:**	
**Hepatitis B** **Hepatitis C** **AIDS** **Osteoporosis** **Diabetes Mellitus** **Hypothyroidism** **Cardiac disease** **Psychiatric disorder** **Hypogonadism**	7(2.8) 65(26) 0 51(20.4) 33(13.2) 34(13.6) 33(13.2) 9(3.6) 112(44.8)

**TableIII:** Comparison of the results of the QOL items and domains between patients and controls

**Domain or item**	**Patient**	**Control**	**P- value**
**Overall QOL**	3.46(SD=0.901)	3.75(SD=0.845)	0.036
**Overall health**	3.33(SD=1.017)	4.10(SD=0.831)	<0.001
**Physical**	64.54(SD=19.028)	71.90(SD=15.374)	0.01
**Psychologic**	55.14(SD=18.77)	63.06(SD=20.24)	0.007
**Social relationship**	59.61(SD=21.17)	67.69(SD=18.47)	0.012
**Environment**	55.84(SD=18.95)	65.00(SD=16.68)	0.001

Note: Item (eg; overall QOL and health) score is 1-5: domain (eg; physical, psychologic, social relationship and, environment) score is 0-100. Higher score indicate better QOL. SD= standard deviation

**TableIV:** Beta coefficients of demographic and medical characteristics associated with QOL domains in regression analysis

**Variable**	**Ferritin**	**Education**	**Sex**	**Hepatitis C**	**Cardiac disorders**	**Psychiatric disorders**	**Splenectomy**	**Chelator**	**Age**
**Domain**									
**Overall QOL**	-0.07	0.35^a^	0.12	0.26^a^	0.43^a^	0.81^a^	0.02		
**Overall health**	-0.13^a^		0.22	0.38^a^	0.66^a^				
**Physical**		6.60^a^			11.8^a^				-0.53^b^
**Environmental**		8.80^a^				18.37^a^			
**Social relationship**		6.33^b^				12.4		-2.47^a^	-0.62^a^
**Psychological**		6.30^a^		6.53^a^	8.49^a^				

## Discussion

In comparison with previous investigations, this study is the largest study using WHOQOL- BREF for the evaluation of the QOL in patients with thalassemia major (5, 13). Despite the differences between current study with others based on methodology, questionnaire and the number of patients (5, 14-16), we showed similar results and all aspects of the QOL in our patients were impaired. Also, overall health and environment domains were severely affected.

Our results for both univariate and multivariate analyses showed that age was correlated with social relationship and physical domain scores which were inconsistent with studies suggesting that age had no effect on the QOL (7, 15, 17).

In univariate analysis, in agreement with that reported by Donna et al (7),Shaligram et al (15) and Messina et al (17), gender was correlated with overall QOL and overall health scores, but it lost the correlation in multivariate analysis. 

One of the most powerful parameters which correlated with many aspects of QOL was education level, both in univariate and multivariate analyses. In the present study, our patients came from different urban areas of the capital city of Tehran,Iran, and Iran is a developing country with a religious background. In this relatively homogenous community, in terms of ethnicity and religion and social affairs, education is a value. Because it is generally accepted that higher educational level is associated with higher social level and higher income and. So it is not surprising that educational level can be effective in many aspects of the QOL in our study.

Some studies suggested that deferroxamine correlated with lower QOL level (13, 17-20), and oral iron chelator might improve it.(13,18,21-25).Also, in our study, deferroxamine correlated with lower social relationship.

In univariate analysis, ferritin level as an indicator of iron chelation efficacy was correlated with overall 

QOL and overall health items but in multivariate analysis, the correlation existed only with the overall health. This was inagreement with Telfer et al (3). Despite its limitations, higher ferritin level might be indicative for serious iron deposition in vital organs such as heart, pancreas, thyroid and their attendant complications that impacted on overall health item. Lack of correlation of ferritin level with all other aspects of QOL was inagreement with Andreou et al (5).

Psychiatric and behavioral disorders were common in patients with thalassemia major and some studies demonstrated that 80% of these patients at least suffered from one psychiatric disorders(16,26) Also, depression had been listed as a major cause of morbidity in beta- thalassemia(16,27-30) and the prevalence of psychological disorders is reported 40% in previous studies.

In this study, history of psychiatric disorders correlated with 2 of 6 domains of QOL; overall QOL and environment. With optimistic estimations (15, 31), we expected to have at least 100 patients with history of psychiatric disorders, but only nine patients had such histories. On the other hand, psychological domain in our patients was significantly ( P- value = 0.007) lower than controls. It seems that some factors such as cultural issues, lack of paying attention to the sign and symptoms of the psychiatric disorders by patients, parents, and medical staffsand lack of a suitable strategy in national thalassemia program might resulted in this conflicting findings.

## Conclusion

In this study, we showed that all aspects of QOL were impaired in our patients in comparison with their age- sex matched controls. Educational level and disease complications such as cardiac disease and hepatitis Cwere associated with many aspects of QOL. Age, type of chelator, ferritin leveland history of psychiatric disorders were correlated with some aspects of QOL.

These findings were important for future refinement of national thalassemia program. So, we recommended regular screening for psychiatric disorders and facilitated access to oral iron chelators especially defrasirox. Regular monitoring and treatment of complications especially cardiac disease and hepatitis along with strict quality control of blood products are also mandatory. Also, it seems that higher education of the patients may improve quality of life.
